# Curcumin as Prospective Anti-Aging Natural Compound: Focus on Brain

**DOI:** 10.3390/molecules26164794

**Published:** 2021-08-07

**Authors:** Tarek Benameur, Raffaella Soleti, Maria Antonietta Panaro, Maria Ester La Torre, Vincenzo Monda, Giovanni Messina, Chiara Porro

**Affiliations:** 1 Department of Biomedical Sciences, College of Medicine, King Faisal University, Al-Ahsa 31982, Saudi Arabia; tbenameur@kfu.edu.sa; 2 Univ Angers, Université de Nantes, Inserm, CRCINA, SFR ICAT, F-49800 Angers, France; raffaella.soleti@univ-angers.fr; 3 Biotechnologies and Biopharmaceutics, Department of Biosciences, University of Bari, 70125 Bari, Italy; mariantonietta.panaro@uniba.it; 4 Department of Clinical and Experimental Medicine, University of Foggia, 71121 Foggia, Italy; ester.latorre@unifg.it (M.E.L.T.); mondavincenzo@gmail.com (V.M.); Giovanni.messina@unifg.it (G.M.); 5 Unit of Dietetic and Sport Medicine, Section of Human Physiology, Department of Experimental Medicine, Luigi Vanvitelli University of Campania, 81100 Naples, Italy

**Keywords:** curcumin, natural flavonoid, anti-aging, neuroinflammation, telomerases, antioxidant, anti-inflammatory

## Abstract

The nutrients and their potential benefits are a new field of study in modern medicine for their positive impact on health. Curcumin, the yellow polyphenolic compound extracted from *Curcuma longa* species, is widely used in traditional Ayurvedic medicine to prevent and contrast many diseases, considering its antioxidant, immunomodulatory, anti-inflammatory, anti-microbial, cardio-protective, nephron-protective, hepato-protective, anti-neoplastic, and anti-rheumatic proprieties. In recent years, the investigations of curcumin have been focused on its application to aging and age-associated diseases. Aging is a physiological process in which there is a decreasing of cellular function due to internal or external stimuli. Oxidative stress is one of the most important causes of aging and age-related diseases. Moreover, many age-related disorders such as cancer, neuroinflammation, and infections are due to a low-grade chronic systemic inflammation. Curcumin acting on different proteins is able to contrast both oxidative stress than inflammation. In the brain, curcumin is able to modulate inflammation induced by microglia. Finally in brain tumors curcumin is able to reduce tumor growth by inhibition of telomerase activity. This review emphasizes the anti-aging role of curcumin focusing on its mechanism to counteract aging in the brain. Moreover, new formulations to increase the bioavailability of curcumin are discussed.

## 1. Introduction

Nowadays, human life expectancies are increasing, and studies on aging biology are looking to elucidate the biochemical and genetic processes that lead to aging over time and to find new strategies to counter this process.

Aging is a process in which there is an irreversible and progressive decline of physiological functions; this loss could lead to the most important age-related diseases, such as cardiovascular diseases, musculoskeletal disorders and arthritis, neurodegenerative diseases, and cancer [1].

Different aging mechanisms have been identified, including genomic instability, telomere shortening, epigenetic changes, mitochondrial dysfunction, cellular senescence, stem cell exhaustion, and altered intercellular communication [2].

Numerous studies have been published over the past years on nutrition and its impact on health. Several studies have reported that a diet rich in antioxidants and anti-inflammatory may decrease age-related cognitive decline and the risk of developing various neurodegenerative diseases.

**Figure 1:**
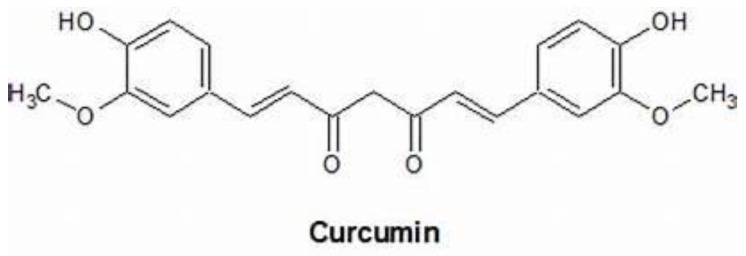
Chemical structure of Curcumin.

Curcumin is a natural dietary polyphenol extracted from *Curcuma longa* Linn with different biological and pharmacological properties including antioxidant, immunomodulatory, anti-inflammatory, anti-microbial, cardio-protective, nephro-protective, hepato-protective, anti-neoplastic, anti-rheumatic, and anti-aging [3]. The chemical name of curcumin is 1,7-bis(4-hydroxy-3-methoxyphenyl)-hepta-1,6-diene-3,5-dione with a chemical formula of C_12_H_20_O_6_; it is formed by two aromatic rings with a methaxy phenolic group, kinked with a linear carbon chain, with an α,β-unsatured β-diketone moiety [4] (Figure 1).

Curcumin, like other polyphenols, has pleiotropic activity. Indeed, due to its capacity to interact with many proteins, curcumin can induce cellular response to external stimuli. Moreover, curcumin up- and downregulates different miRNA and takes part in epigenetic changes in cell [5].

Ageing is one of the risk factors for some types of tumors, and cancer rate is higher in the older compared to younger age categories.

Different factors could explain the link between cancer and aging: during the aging process, there is an increasing of oxidative stress and DNA damages and cell senescence; a progressive decay of immune function occurs in older individuals, and immune response against developing tumors may fail [6].

In this review, our goal is to elucidate the anti-aging proprieties of curcumin in the brain by acting on different target proteins, inducing antioxidant and anti-inflammatory events, modulating microglia neuroprotection, and finally acting on telomerases to arrest cancer progression. Furthermore, we will analyze how to overcome some limitation of clinical application of curcumin represented by poor bioavailability low solubility and stability related to its hydrophobic structure with innovative biotechnological strategies, such as the nanodelivery-based approaches.

## 2. The Biology of Aging Process, Its Hallmarks and Biomarkers

Aging is a complex process derived from the interaction of different events, including random, environmental, genetic and/or epigenetic interfering with body functions [7,8]. Aging is characterized by a declined physiological function affecting most living organisms, which is underpinned by alterations within molecular pathways and is also the most profound risk factor for a large number of premature age-associated diseases. Moreover, aging is associated with multifaceted changes involving all the levels of the human body organization. This includes neurodegenerative, musculoskeletal, metabolic, cardiovascular, immune system disorders, and cancer that may increase the vulnerability to death [1,9,10].

Among the commonly described cellular and molecular aging hallmarks are genomic instability, telomere attrition, epigenetic alterations, loss of proteostasis, deregulated nutrient-sensing, mitochondrial dysfunction, cellular senescence, altered intercellular communication, and stem cell function decline [2,11,12,13]. Indeed, the regenerative and repair potential of many tissues decline with age due to the reduced ability of several stems cells to repair tissues [14]. Consequently, the transplantation of aged hematopoietic stem cell transplantation would be compromised. Despite the existing data, there is no conclusive evidence about which molecular, cellular, or physiological changes are the most important drivers of the aging process and/or how they influence each another [15,16].

In spite of the existing conserved hallmarks/markers of aging, the consequences of aging can vary not only among a single individual tissues but also among individuals. Although the various molecular causes of aging with highly complex interactions described in the literature, understanding the fundamental mechanisms for many pathways remains not fully understood.

## 3. Antioxidant Role of Curcumin

It has been widely described that increased oxidative stress altered the structure and functions of lipids, proteins, and nucleic acids, thereby contributing to the accumulation of dysfunctional proteins and lipid peroxidation. Damaged nuclear and mitochondrial DNA leads particularly to mitochondrial dysfunction and cell death [17,18]. In turn, these dysfunctions not only speed up the body aging process but ultimately contribute to the development of a wide variety of both chronic and degenerative disorders, such as neurodegenerative diseases (Alzheimer’s and Parkinson’s disease), dementia, cancer, atherosclerosis, obesity, diabetes, vascular diseases, osteoporosis, metabolic syndrome, and aging [19,20].

Indeed, the damage caused by oxidative stress is an important hallmark of aging and considered as an essential component of the pathogenesis pathways of multiple age-related diseases as well as the disease status [21]. Furthermore, the oxidative stress is caused by an imbalance between the production of reactive oxygen species (ROS) in cells and tissues and the ability of biological systems to detoxify these reactive products [22]. Importantly, the aging process may be corrected by environmental, pharmacological, and nutritional strategies [23].

Remarkably, investigating the role of natural substances such as curcumin or derivatives with high antioxidant potential that counteract oxidative stress seems to be an effective preventive measure against free radical-linked aging [24] (Table 1). This would provide an evident approach for potential therapies that can promote healthy aging.

A large number of studies highlighted the protective effect of curcumin against oxidative and nitrosative stress in multiple cellular and animal models. This effect is achieved through the reduced levels of malondialdehyde (MDA), protein carbonyls, thiols, and nitrotyrosines [25]. Additionally, curcumin stimulated the activities of superoxide dismutase (SOD) and the catalase, the key antioxidant enzymes of the defense mechanisms against the free radicals produced during metabolic reactions [26].

The oxidative stress can be reduced by three main strategies: (1) reducing exposure to environmental factors; (2) lowering the oxidative stress by stabilizing mitochondrial energy production and efficiency; (3) increasing the endogenous and exogenous antioxidants levels [27]. Physical activity is one of these strategies known to counteract the negative effects of oxidative stress and delay aging. Indeed, exercising at a moderate-to-vigorous intensity for at least 5 days per week, along with a proper lifestyle are critical elements to counteract the toxicity and harmful effects of oxidative stress on health by increasing antioxidant levels [28]. In addition, moderate and regular physical exercise has been reported to be therapeutic in aging and reduce the risks of a great number of age-related diseases. Despite these health-promoting effects, a single bout of physical exercise can increase metabolism, oxidative stress, inflammation, and muscle fatigue immediately after exercising [29].

Nutraceutical formulations have been shown to have anti-aging role, and their consumption is highly recommended as a preventive antioxidant tool, together with a constant and adequate physical activity [30,31]. Due to its chemical structure, curcumin has proved to be an excellent scavenger of ROS and reactive nitrogen species (RNS) [32] and is able to attenuate or prevent the exercise-induced oxidative stress and inflammation, by modulation of GSH, catalase, and SOD enzymes and inhibiting of ROS-generating enzymes such as lipoxygenase/cyclooxygenase and xanthine hydrogenase/oxidase [31]. This has strengthened our conviction that curcumin is the golden nutraceutical with proven potential in preventing/delaying the onset of age-related diseases [33,34].

Randomized controlled trials conducted ≥4 weeks investigating the effects of curcumin supplementation on oxidative stress biomarkers, including glutathione peroxidase (GPX) activity in erythrocytes, serum MDA concentrations, and SOD activity have shown a significant reduction in circulating level of MDA and a significant increase in SOD activity. This lowering effect was observed at curcumin doses ≥600 mg/day [35].

A large body of evidence suggests that oxidative stress promotes the development of ovarian aging and its aging-associated disorders, including telomere shortening, mitochondrial dysfunction, apoptosis, and inflammation. This results in age-related fecundity decline in human and diverse animals [36]. Curcumin showed a protective effect in the ovaries involving multiple mechanisms [37]. The specific effects and mechanisms involved the following mechanisms: (1) alleviating ovarian oxidative injury, increasing nuclear factor-erythroid-2-related factor 2 (Nrf2), heme oxygenase-1(HO-1), SOD, and SOD1 levels while reducing ROS production and MDA levels; (2) decreasing the levels of caspase-3 and -9; and (3) as an anti-inflammatory agent, reducing the inflammatory markers levels such as CRP, TNF-α, and IL-6. These findings suggest that curcumin as oxidative stress modulator may represent a therapeutic intervention for delaying ovarian aging [37,38,39,40,41,42].

As discussed above, aging is associated with various changes in organ structure and function. Thus, renal aging is multifactorial and complex process characterized by many morphological and functional changes. The factors involved in renal aging include telomeres shortening, cell cycle arrest, chronic inflammation, activation of renin-angiotensin aldosterone system, reduced antioxidant capacity, and the development of glomerular fibrosis. Curcumin displays potent biological and pharmacological effects on renal health [43]. Aging is an independent risk factor increasing the likelihood of developing cardiovascular diseases which is due primarily to the arteries remodeling and the development of vascular endothelial dysfunction [44]. Another promising anti-aging potential of curcumin supplementation was shown in healthy middle-aged older men and postmenopausal women. Indeed, 12 weeks curcumin administration has improved resistance artery endothelial function by increasing NO bioavailability and reducing vascular oxidative stress. This suggests the critical role of curcumin to maintain health vascular endothelium with aging, a fundamental element in the prevention of atherosclerosis and arterial diseases [45]. Another study provides additional support about the role of curcumin associated with aging in patients at risk of cardiovascular diseases through reducing serum LDL-cholesterol and triglyceride levels [46]. Determining the long-term benefits of curcumin in patients with cardiovascular diseases or at risk to develop cardiovascular disorders seems like a promising research avenue. The accelerated aging induced by oxidative stress results in sex-specific differences in longevity and susceptibility to age-related neurodegeneration. In a previous research, curcumin was shown to prolong lifespan of fruit fly model (*Drosophila melanogaster*) through enhancing SOD activity [47]. These findings were corroborated by other data where curcumin induced sex-specific in vivo responses to oxidative stress. This includes protection from hydrogen peroxide and alterations in behavior of *Drosophila melanogaster*. This may rely on gene expression and support the anti-aging role of curcumin in gender-dependent manner [48]. Curcumin belongs to the class of hormetic agents that stabilize Nrf2 and enhance expression of HO-1. Curcumin triggers Nrf2 pathway, which has a pivotal role in activating antioxidant enzymes, such as thioredoxin reductase, Hsp70, sirtuins [49,50,51,52]. Furthermore, another study finding reported that curcumin increased the activity of several antioxidant enzymes including protein thiol, non-protein thiol, GPx, and SOD in dogs fed with curcumin on day 30 compared with control dogs. In addition, curcumin consumption stimulated the antioxidant capacity in the serum of dogs and consequently reducing ROS levels. Curcumin improved animal health, with particular emphasis on the stimulation of the antioxidant system and evidence of an anti-inflammatory effect. This suggested that curcumin exerts beneficial effect on both growth, health and consequently slowing down aging [53].

**Figure 2:**
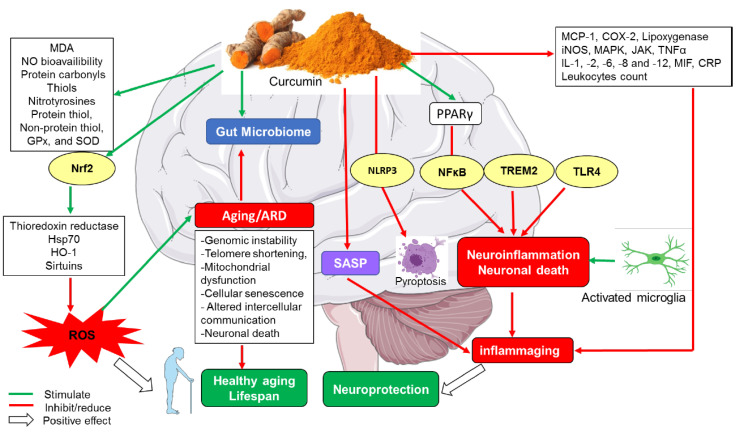
Neuroprotective and anti-aging-effects of curcumin.

Curcumin supplementation accompanied with regular physical exercise could potentially slowing down aging and/or preventing oxidative stress-induced age-related functional and structural changes and the age-related disorders. Collectively, these findings reinforce the antioxidant potential of curcumin on organ health function in the context of aging (see Figure 2). Further investigations are warranted to unravel the exact molecular targets and signaling pathways responsible for the antioxidant effects of curcumin in different human populations.

## 4. Anti-Inflammatory Role of Curcumin

Inflammation is one of the leading causes of aging, which is often associated with an impaired healing process [54]. Particularly, low-grade inflammation is believed to contribute substantially to aging process and results in various aging-related decline in many organ functions [55,56]. Of particular interest, ageing is characterized by an increased circulatory level of pro-inflammatory mediators, a phenomenon that has been termed “inflammaging”.

Furthermore, gut microbiota and diet were shown to influence low-grade inflammation. Recent findings suggest dietary interventions, including curcumin supplementation, as a strategy to combat inflammaging. Interestingly, the age-modulatory properties and healthful effects of curcumin have been illustrated in different cellular and animal models, including *C. elegans*, Drosophila, and mice. As it was clearly discussed above, curcumin was found to extend both healthspan and lifespan, mainly blocking the most relevant proinflammatory pathway NF-kB [57] (Table 1).

In addition to the well-documented evidence supporting the numerous biological properties of curcumin in inhibiting NF-κB signaling dependent inflammation [34,58]. another further implication in reducing the intensity of inflammaging has been described. Indeed, curcumin was shown to modulate the senescence-associated secretory phenotype (SASP), which characterizes senescent cells and contributes to fuel the inflammaging [59,60].

Interestingly, the short-term treatment of cells with low concentrations of curcumin decreased the level of secreted pro-inflammatory cytokines such as IL-8 in normal young cells [61]. Moreover, lower doses of curcumin have increased the production of sirtuin, i.e., NAD-dependent deacetylases, and sirtuin 1 reduced inflammation by inhibiting NF-κB signaling [62]. It is believed that curcumin exerts its effect in dose-dependent and cell-context manner on the protein activity involved SASP.

Particularly, increasing evidence suggests that repeated stimulation of innate immune responses over time [63] results in the development of inflammaging. In these settings, both an increased burden of senescent cells during aging and a hyper-stimulation of macrophages over time can play key roles of inflammaging process.

Recent reports of randomized controlled trials conducted from 2008–2020 have demonstrated that curcumin was able not only to modulate the antioxidant status but also restore quantity, quality, and functional-metabolic status of immune cells. This lends support to other data showing partial anti-inflammatory, immunotropic and antioxidant activity of turmeric extract in vitro and in vivo. Further implication of curcumin in modulating aging-related inflammation through lowering CRP level in dose-dependent manner in rats’ model was reported. Moreover, MDA and NO levels were increased significantly in animals fed with curcumin [64]. This has strengthened our belief that curcumin slows down the aging process by suppressing age-related inflammatory indices.

In addition to the role of NF-kB signaling pathway in inflammatory process, circulating levels of MCP-1 were found to increase with aging and considered as potential aging biomarker [65,66,67]. Interestingly, anti-inflammatory effects of curcumin have been shown to encompass the inhibition of MCP-1 [33]. Other anti-inflammatory effects involved the downregulation of inflammatory mediators such as COX-2 activity, lipoxygenase, iNOS, MAPK, JAK and inhibition of TNF-α production, IL-1, -2, -6, -8, and -12, macrophage migration inhibitory factor (MIF) [66].

A recent study has shown that curcumin not only stimulated the antioxidant system and reduced oxidative reactions in dogs but also reduced leukocyte counts, which suggests mild anti-inflammatory effects achieved in dogs fed with at a dose of 30 mg of curcumin/dog/day [53]. These substantiate previous findings [67], where it was observed that nursing lambs fed with curcumin had lower total leukocytes, neutrophils, and lymphocytes. A similar effect was reported in rats treated with 50 and 400 mg/kg curcumin, indicating a remarkable improving effect on health and the immune response [68]. This points toward the importance of curcumin in reversing the inflammatory responses and enhancing the immune system performance, both playing a critical role in ameliorating health and consequently slowing down aging (see Figure 2).

## 5. Neuroprotective Role of Curcumin

Advanced age is considered a major risk factor of cognitive dysfunction and neurodegenerative diseases. Cellular senescence stimulates the secretion of proinflammatory cytokines that cause chronic inflammation regardless of the activation of the immune system. This phenomenon of chronic system inflammation that accompanies aging is called ‘inflammaging”, resulting in death and cognitive decline [69,70,71,72,73]. Among its multiple properties, curcumin is also known for its anti-protein-aggregate and neuroprotective activities improving the prognosis of neuro-inflammatory diseases that we have previously discussed [4,74] (Table 1).

However, the major obstacle for curcumin delivery into the brain is the blood–brain barrier (BBB) [75]. Potential clinical applications of nano-curcumin are emerging and able to overcome therapeutic obstacles of free curcumin and ameliorate many aging-related cellular and organ dysfunctions [76].

Aging could dramatically alter gut microbiome and lead to deleterious changes to the gut-brain axis [77] including endocrine, nutrient, immunological and neural signals between gut and brain via enteric nervous system (ENS) and consequently to multiple central nervous system (CNS) disorders such as multiple sclerosis, depression, anxiety [78]. In addition to the development of various degenerative disorders including AD, PD, multiple system atrophy (MSA), neuromyelitis optica (NMO), and amyotrophic lateral sclerosis (ALS) [79] as we age, these perturbations might also be triggered indirectly by health status, increasing need for medications such as: NSAIDs, antibiotics and malnutrition [80,81]. As the gut–brain axis is linked to neurodegeneration, curcumin exerts neuroprotective effect against neurodegenerative disorders by restoring the intestinal barrier function and a healthy gut microbiome [82].

The investigation of curcumin effects on diabetic rats’ brains has demonstrated that curcumin or curcumin analogue A13 treatment reduced inflammation through inhibiting NF-κB p65 canonical pathway and decreasing the level of TNF-α and Cox-2 in the in diabetic rats’ cerebral cortex. Curcumin and A13 decreased oxidative stress through increasing the activity of SOD and decreased the malondialdehyde MDA level in the brain of diabetic rats [83]. These findings highlight the importance of neuroprotective effect of curcumin against the brain damage in diabetic rats by regulating both inflammation and oxidative stress. This is consistent with previous findings where curcumin was shown to reduce significantly the mRNA expression of NF-kB and TLR4 and showed protective effects against glutamate neurotoxicity in the male albino rats [84].

Interestingly, another study analyzing the curcumin-mediated neuroprotective effects on brain aging induced by d-galactose in in vitro and in vivo models revealed an anti-aging effect through regulating neuronal loss, apoptosis in D-galactose induced brain aging, and anti-oxidant enzyme expression. [85].

Furthermore, curcumin improved neuronal length and cellular senescence down-regulated expression of p16 and p21 and upregulated expression of antioxidant enzymes, including SOD-1, GPX-1, and catalase. Administration of curcumin ameliorated the cognitive impairment and suppressed apoptosis in the cerebral cortex by downregulating Bax and poly (ADP-ribose) polymerase expression and increasing Bcl-2 expression [86]. In neurodegenerative diseases, such as AD, PD, ALS, microglia play an important role by inducing oxidative stress, redox imbalance and neuroinflammation. The activated microglia are represented by M1 (pro-inflammatory) and M2 (anti-inflammatory) functional phenotypes based on the surface molecules and cytokine expression profiles. Different natural products show therapeutic properties on microglia and consequent prevent neurodegenerative diseases; they act by inhibition of microglia polarization and production of inflammatory mediators. In microglia, curcumin acts on different molecular targets. Curcumin inhibited LPS-induced NF-kB and activator protein-1 (AP-1) DNA bindings in BV2 microglial cells [87] decreasing inflammatory mediators. Peroxisome proliferation-activated receptor-γ (PPARγ) is a transcription factor and nuclear receptor protein that regulates inflammatory responses in microglia, astrocytes [88] and when is activated, PPARγ suppresses the production of proinflammatory cytokines and inflammatory pathways by binding the peroxisome proliferator response element [88]. Curcumin activates PPARγ which reduces NF-κB cytokine production in a mouse model of AD, in rat hippocampal primary cell lines and primary astrocytes [89]. Moreover, our group has found that curcumin suppresses LPS induced inflammatory response in microglia cells by down regulation of PI3K/Akt [90,91] and JAK/STAT/SOCS signaling pathway [92]. In addition, curcumin induces anti-inflammatory mediators, such as HO-1/NRF-2 consequently reducing oxidative stress and neuroinflammation [93]. Curcumin treatment improved neuron loss and degeneration, while also inhibited cellular senescence and oxidative stress by upregulating antioxidant enzyme expression in RA-induced SY5Y cells [94]. In line with the findings described above, the protective effect of curcumin against cognitive impairment has been demonstrated in diabetes mellitus/chronical cerebral hyperperfusion-induced cognitive deficit model. Moreover, curcumin treatment attenuated the neuronal death and suppressed neuroinflammation induced by microglial activation [95]. These protective effects involved the modulation of triggering receptor expressed on myeloid cells 2 (TREM2)/TLR4/NF-kB pathway. Curcumin treatment reduced nod-like receptor protein 3 (NLRP3) dependent pyroptosis. [95]. Since NLRP3-dependent pyroptosis has been reported to be involved in the progression of neurodegenerative diseases, this result suggests that curcumin may be useful as pharmacological strategy for neurodegenerative diseases. Further studies are needed for better understanding of curcumin’s promising effects in preventing the neuronal loss and cognition-decline related to aging [96].

**Table 1:** Curcumin properties.

Properties	Mechanisms	References
Antioxidant	↓ MDA level ↑ Antioxydant enzyme activities ↑ NO bioavailability ↑ ROS and RNS scavenging	[25,35,64,83] [26,31,37,47,49,50,51,52,83,86,93] [45,64] [32]
Anti-inflammatory	↓ MCP-1, CRP, TNF-α, IL-6, IL-8, MIF expression ↓ NF-kB pathway ↓ SASP activity ↓ COX-2, Lipoxygenase, iNOS, MAPK, JAK activities ↑ PPARγ pathway ↓ NLRP3 activity	[33,41,61,64,66] [57,58,62,87,89] [59,60] [66,92] [88,89] [95]
Neuroprotective	↓ Neurotoxicity ↓ Neuronal loss ↓ Apoptosis ↓ Senescence ↓ Microglia and astrocyte inflammation ↓ Pyroptosis	[84] [85,94] [85,86,94,95] [86,94] [87,88,89,92,95] [95]

## 6. Curcumin and Telomerases in Brain

ROS induced oxidative stress, known as a potential contributor to the aging process which arise as damages caused by products of energy metabolism in the mitochondria [97] and in turn lead to telomere shortening.

Telomerase is present in almost all eukaryotic organisms and was studied firstly in protozoans by Nobel Laureate Elizabeth Blackburn, its importance for human health during development, ageing and cancer soon became obvious [98].

Telomers are highly conserved repetitive DNA sequences located at the end of chromosomes, that control the cell replication and contribute to maintain chromosomal stability. Telomers decrease of 50–200 bases after each round of cell division. When telomere reaches a critical minimum length, the cells become senescent. Dividing cells express telomerase, a ribonucleoprotein enzyme that synthetizes and elongates telomeric DNA [99].

Human telomerase contains two subunits: human telomerase RNA component (hTR; also known as hTERC) and human telomerase reverse transcriptase (hTERT). hTR is composed by an RNA template complementary to the 3′ overhang of telomeres [100]. hTERT acts as the catalytic subunit that adds telomeric DNA to the 3′ overhang [101,102].

The expression level of *hTERT* mRNA highly correlates with cellular telomerase activity [103], indicating that hTERT is essential for telomerase activity. Thus, it will be useful study the mechanism that underlying hTERT regulation in order to take advantage of telomerase for cancer diagnosis and treatment.

Telomerase is present in ~90% of cancer cells and tumor tissues, demonstrating that they contribute to the infinite proliferation of cancer cells [104]. Curcumin was shown to have inhibitory effects on telomerase and induced telomere shortening and apoptosis in brain tumor cells. Curcumin induced growth inhibition and cell cycle arrest at G2/M in medulloblastoma and glioblastoma cells [105].

In various types of cancers, curcumin was shown to selectively target cells that express telomerase enzyme making these cells more vulnerable to curcumin-induced cytotoxicity of cancer cells. Importantly, the above-mentioned study revealed that the complex and diverse action of curcumin, and its efficacy could depend on the cell types used. The long-term studies on brain tumor cells highlighted the use of curcumin as an adjuvant for cancer therapy. Telomere shortening drives renal cell senescence and leads to renal aging.

Khaw and co-workers have demonstrated that curcumin suppresses telomerase activity in brain tumor cells which is associated with reduction in hTERT levels. Treatment with curcumin induces a significant telomere shortening in brain tumor cells suggesting its potential clinical application as telomerase inhibitor and use of curcumin in adjuvant cancer therapy [105]. By contrast, in normal cells curcumin improves viability by acting on telomerase when the cells have been stimulated with toxic molecules. A study conducted on SK-N-SH cells treated with Aβ1–42, curcumin, and Cur1 improved cell viability. Normally, hTERT was inhibited by Aβ1–42; shortened telomere could not restore length, and then, there were plenty of apoptotic cells. Treatment with curcumin and Cur1 could bind to Aβ1–42 and antagonize neurotoxicity; thus, the expression of hTERT was upregulated, shortened telomere restored length and the numbers of cells were increased. Upregulation of hTERT was not observed in curcumin or Cur1 treated SK-N-SH cells without Aβ1–42 treatment leading to the conclusion that curcumin and Cur1 have no effect on hTERT upregulation in normal cells [106]. Importantly, the action of curcumin is complex and different, and its efficacy may depend on the cell types used in the study. Long-term studies on brain tumor cells underscore the use of curcumin in adjuvant cancer therapy.

## 7. New Nanodelivery Strategies to Increase Pharmacological Activities of Curcumin

To increase the solubility, stability, bioavailability, and activity of curcumin, a common strategy has been found in different research: the encapsulation.

Several research groups showed the improvement of encapsulated curcumin compared to free molecule. Two major class of nanocarriers have been used: synthetic and natural nanocarriers.

Different types of synthetic nanocarriers have been developed to deliver curcumin: lipid-based curcumin formulations (liposomes, solid-liquid nanoparticles, nanostructured lipid carriers) and polymeric-based curcumin formulations (micelles, polymeric nanoparticles, polymeric conjugates) (for review see [96,107]).

**Figure 3:**
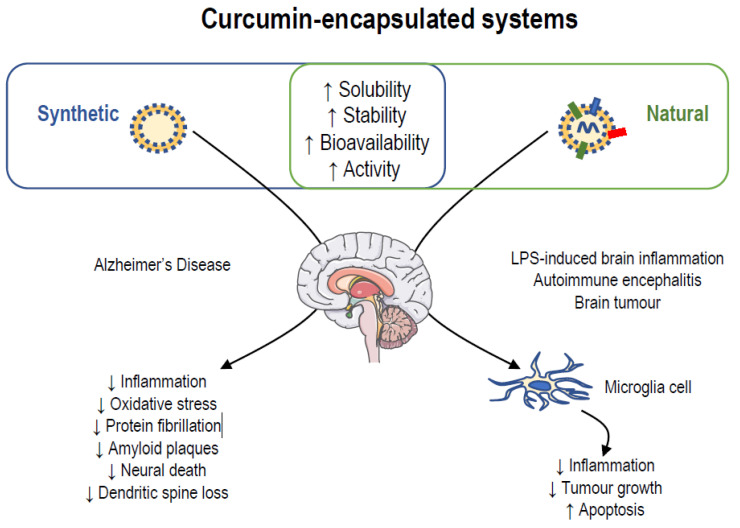
Curcumin-encapsulated systems in the brain.

Due to the richness of works focused on strategies using these synthetic curcumin formulations, we will summarize below only two strategies used for its nanodelivery (See Figure 3).

Liposomes are systems composed of single or multiple bilayers made of phospholipids which entrap hydrophilic, lipophilic, and amphiphilic molecules [108]. Modifications of this conventional structure have been elaborated, such as liposomes containing a surface layer of polyethylene glycol, theragnostic liposomes containing image agent, and liposomes containing specific target ligand [109]. In vitro, liposomal curcumin resulted in concentration-dependent inhibition of proliferation, induction of apoptosis, and suppression of motility of endometrial carcinoma cell [110]. Moreover, no demonstrable toxicity has been found in the zebrafish model, and tumors are suppressed after treatment with curcumin-encapsulated liposomes [110].

Moreover, the reduction of the dysfunctions observed in neurodegenerative diseases has been evidenced using solid lipid curcumin particles. Acute treatment of solid lipid curcumin particles provides more anti-amyloid, anti-inflammatory, and neuroprotective effects than free curcumin in a mouse model of Alzheimer’s disease [111]. In the same animal model, solid lipid curcumin particles decreased amyloid plaques and neuronal death, prevented dendritic spine loss, and preserved pre- and postsynaptic markers, along with partially improving behavioral outcomes [112].

Micelles are self-assembling nanosized colloidal particles with a hydrophobic core and hydrophilic shell [113]. It has been shown that curcumin micelles are an excellent intravenously injectable aqueous formulation of curcumin; this formulation can inhibit the growth of colon carcinoma through inhibiting angiogenesis and directly killing cancer cells [114].

Moreover, it has been noted that curcumin-loaded nanomicelles suppressed the AD progression reducing protein fibrillation and inhibiting the amyloidogenesis through glycation process due to curcumin release, thus preventing the formation and accumulation of amyloid fibrils and glycation. This effect is also sustained by higher effectiveness of curcumin-loaded micelles due to their degradation or hydrolysis and subsequently the release of curcumin as an antioxidant agent [115].

In a recent study evaluating the comparative effect of conventional curcumin with curcumin nanoparticles—liposomal Curcumin (LCC)—on an experimental rat’s model of Gentamicin induced nephrotoxicity, it was observed that LCC was more efficient. Interestingly, LCC improved all the oxidative stress parameters: MDA, NO, total oxidative stress. Taken together, curcumin showed a dose-dependent improving effect on plasma oxidative stress parameters/antioxidant capacity, MMP-2 and -9 level, and renal function parameters in Gentamicin-induced nephrotoxicity model [116].

Among natural nanocarriers, the exosomes have been used as efficient drug delivery system [117,118]. Exosomes belong to extracellular vesicle family and are released from cells by exocytosis after the maturation of multivesicular bodies. Their protein, lipid, and nucleic acid composition confers them the ability to mediate cellular communication. Exosomes possess the intrinsic property to be biocompatible, and they do not cause any side-effects. In addition, their small size allows to cross biological barriers and to escape immunity system. They can bind hydrophobic molecules, like drugs, favoring their transport, bioavailability, and uptake [117]. Indeed, exosomes can be manipulated in order to generate curcumin-encapsulated exosomes. Two strategies of passive encapsulation are possible: (i) cell treatment with curcumin and isolation of released exosomes (loaded exosomes) and (ii) load curcumin in the exosomes (primed exosomes).

Curcumin can be self-assembled into the lipid membrane of exosome by the interaction between the hydrophobic tails and hydrophobic drug. The insertion into lipid bilayer ensured protection of curcumin from degradation [119].

The therapeutic effect of loaded-curcumin exosomes was first demonstrated in a context of inflammation [119]. Encapsulated curcumin increased the solubility, stability, and bioavailability of curcumin and enhanced delivery of curcumin to activated monocytes. As a consequence, this novel drug delivery system ensured protection of mice from LPS-induced septic shock [119].

Other studies demonstrated the beneficial effect of exosomes produced upon treatment of different kinds of cells with curcumin. It has been showed that curcumin promoted exosome secretion in a model of intracellular cholesterol trafficking impairment [120]. Indeed, loaded exosomes from curcumin-treated leukemia cells decreased leukemic cell growth [121] as well as tumor angiogenesis by decreasing the migration of endothelial cells, the expression of vascular cell adhesion molecule-1, and the reduction of capillary-like structures [122].

Similarly, exosomes from pancreatic adenocarcinoma cells and from non-small cell lung carcinoma treated with curcumin possessed anticancer properties [123,124].

Exosomes from mouse brain endothelial cells treated with curcumin increased junction protein expression and ameliorated endothelial cell permeability. The beneficial effects of these exosomes are also due to their ability to lowering endothelial oxidative stress [125].

Curcumin-loaded exosomes were intranasally administrated in three inflammation-mediated disease models, an LPS-induced brain inflammation model, experimental autoimmune encephalitis, and a GL26 brain tumor model protected from LPS-induced brain inflammation; the progression of myelin oligodendrocyte glycoprotein peptide induced experimental autoimmune encephalomyelitis and had significantly delayed brain tumor growth in the GL26 tumor model without observable side effects [126].

Furthermore, the therapeutic potential of curcumin-loaded embryonic stem cell exosomes in neurovascular restoration following ischemia-reperfusion injury in mice has been noted. Treatment with these exosomes triggered a series of beneficial effects including reduced neurological score, infarct volume, edema, inflammation, and astrogliosis [127].

Exosomes derived from curcumin-treated buffalo granulosa cells alleviated LPS-mediated inflammation by decreasing pro-inflammatory cytokine expression and restoring 17-β estradiol production [128].

Recently, exosomes have been engineered for the pulmonary delivery of therapeutic peptides and curcumin into the lungs by inhalation [129,130]. These exosomes increased curcumin delivery and pro-inflammatory cytokines in LPS- activated cells. In an animal model of ALI, they also increased the delivery efficiency of curcumin, reducing inflammation in the lungs.

All studies suggest that encapsulated curcumin may be considered an excellent tool for the treatment of different pathologies, by increasing bioavailability and efficacy of curcumin without observed side effects.

## 8. Conclusions

The discovery of new strategies to contrast aging and aging-related diseases is an important goal of modern research. In our view, curcumin is one of the best candidates to achieve this goal with its antiviral, antinociceptive, anti-inflammatory, antipyretic, and antifatigue proprieties. It is important to highlight that Curcumin is devoid of any significant toxicity in most of the preclinical as well as clinical investigations, and few investigations have reported negative effects of curcumin. In addition, natural products may be a safe, secure, and dependable source to find drugs responsible for controlling the current pandemic, and even if the beneficial effects of curcumin against SARS-CoV-2 have not yet been reported, curcumin has some useful clinical effects that could be effective to manage the symptoms of the infected patient with COVID-19. Curcumin in fact can modulate the events of SARS-CoV-2 cellular entry, their replication, and molecular cascade manifesting pathophysiological consequences of COVID-19. Due to its important and healthy proprieties, we think that dietary supplementation with curcumin could be a suitable approach to prevent a large panel of diseases and improve the quality of life.

In this review, we have described the anti-aging potential of curcumin with particular regard to prevention and treatment of brain diseases, in different ways: (1) by acting on different target proteins, (2) by inducing antioxidant and anti-inflammatory events, (3) by modulating microglia neuroprotection, and (4) by acting on telomerases to arrest cancer progression.

The new formulations of curcumin discussed in this review may help to improve bioavailability and stability of the natural compound, increasing its anti-aging potential.

This last aspect regarding the anti-aging power of curcumin may increase the range of pharmacological applications of the yellow polyphenol and merits further investigations in in vivo models, as well as in clinical investigations.

## Data Availability

The data presented in this study are available on request from the corresponding author.

## Sample Availability

Samples of the compounds are not available from the authors.
